# Sleep Quality and Associated Factors among Diabetes, Hypertension, and Heart Failure Patients at Debre Markos Referral Hospital, Northwest Ethiopia

**DOI:** 10.1155/2020/6125845

**Published:** 2020-05-21

**Authors:** Afework Edmealem, Sr. Genet Degu, Dessalegn Haile, Mihretie Gedfew, Bekalu Bewket, Atsedemariam Andualem

**Affiliations:** ^1^Department of Nursing, School of Nursing and Midwifery, Wollo University, Dessie, Ethiopia; ^2^Department of Midwifery, Debre Markos University, Debre Markos, Ethiopia; ^3^Department of Nursing, Debre Markos University, Debre Markos, Ethiopia

## Abstract

**Background:**

Chronic illnesses have a negative impact on the quality of sleep; however, patients with chronic illness do not bring sleep issues while they are coming to a health institution for a follow-up. As a result, poor sleep quality among patients with chronic illness is often unrecognized and untreated, and it results to a negative impact on the prognosis of chronic illness.

**Methods:**

An institutional-based cross-sectional study design was employed from February 22, 2018, to April 6, 2018. The total sample size was 396. The study employed a stratified random sampling technique, and study participants were selected by systematic sampling. The data were collected by a Pittsburgh Sleep Quality Index (PSQI) questionnaire which is a validated and standardized tool. The data were analyzed by SPSS version 25; text, tables, and figures were utilized for data presentation. By considering a 95% confidence level and *P* value of 0.05, binary logistic regression and Kruskal-Wallis test were enrolled.

**Results:**

The prevalence of poor sleep quality among diabetes, hypertension, and heart failure patients was 36.5%. The odds of being a poor sleeper are increased when age increased. Patients who have poor perception towards the prognosis of their illness were four times more likely to be a poor sleeper compared to patients with good perception (AOR = 4.21, 95%CI = 1.94‐9.13, *P* = 0.001). Patients who have anxiety were four times more likely to be a poor sleeper compared with patients without anxiety (AOR = 3.69, 95%CI = 2.19‐6.20, *P* = 0.001). The educational level and residence were other factors associated with sleep quality. There was a statistically significant difference of sleep quality between patients with diabetes and hypertension, and diabetes and heart failure (*F* (2, 384) = 10.92, *P* = 0.004). *Conclusion and Recommendations*. In this study, over one-third of patients had poor sleep quality. Age, educational level, residence, perception towards prognosis of illness, and anxiety were factors associated with sleep quality. All health care providers should assess and provide advice about sleep hygiene and influencing factors. Assessment of sleep quality for every diabetes, hypertension, and heart failure patients in every visit should be incorporated in the care package.

## 1. Introduction

### 1.1. Background

Sleep is a basic need which is initiated by a circadian rhythm and sleep/wake homeostatic pressure and followed by a period of wakefulness [1]. Sleep represents an obligatory element for health and well-being which maintains cognitive performance, physiological process, emotion regulation, physical development, and quality of life [2]. It helps to modify body temperature, cardiac work, and hormone production that results to an essential restorative state and proper functioning of the organism [3].

Sleep quality has no clear cut definition although great attention is given since the past few decades. It has 7 dimensions. These include subjective sleep quality, sleep latency (how long it takes to fall asleep), sleep duration, habitual sleep efficiency (the percentage of time in bed that one is asleep), sleep disturbance, use of sleep medications, and daytime dysfunction [4]. According to the 2017 report of the National Sleep Foundation in USA, good sleep quality is indicated by sleeping more time while in bed (at least 85 percent of the total time), falling asleep in 30 minutes or less, waking up not more than once per night, and being awake for 20 minutes or less after initially falling asleep [5].

Adequate quality and duration of sleep like diet and exercise positively influence many aspects of health including physical, cognitive, and emotional health [6]. As to the 2015 National Sleep Foundation Guideline, the recommended sleep duration is 7 to 9 hours for young adults and adults and 7-8 hours of sleep for older adults [2]. Despite this fact, the public attention to sleep quality is low [7]. The quality and duration of sleep are disturbed by crowded urbanization, long work schedule, night and shift work, spending more time in watching television and using internet, and disease conditions [8].

Chronic sleep deprivation is estimated to affect between 7.5 and 20% of the general population [9]; however, patients with chronic illness do not bring sleep issues while they are coming to a health institution for a follow-up [1]. As a result of this, poor quality of sleep among patients with chronic illness is often unrecognized and untreated [10, 11].

Pathological and night time sleep deprivations have substantial adverse effects on regulation of weight, sugar, and blood pressure because of endothelial dysfunction, sympathetic nervous system stimulation, and regulation and activation of systemic inflammation [2, 6] which increases further complication of chronic illnesses such as diabetes, hypertension, and heart diseases [12, 13]. Studies show that poor sleep quality impaired exercise capacity of patients, resulting in adverse prognosis of the disease [12]; impaired functional outcomes [14, 15], daily functions [16], and self-care behavior of patients; and increases of the burden of the disease [10]. All these increase the health care expenditure of one's country and lead to poor control of disease and poor quality of life [1, 17].

Poor sleep quality not only has been associated with various diseases but also leads to occupational accidents [1], poor performance, higher health care utilization, car crash injuries [8], falls especially in older adults [18], and suicidal ideation [19]. Thus, identifying and treating coexisting sleep problems among patients with chronic illness will improve the treatment outcome of patients [20].

Poor sleep quality is a neglected public problem in Ethiopia that lowers the functional outcome of individuals especially individuals with chronic illness. It is an unrecognized and underattention factor that affects self-care behavior and daily function of patients and increases the adverse prognosis of chronic illnesses such as hypertension, heart failure, and diabetes. Despite this fact, there is no adequate study which assessed the quality of sleep among patients with chronic illnesses such as hypertension, heart failure, and diabetes in Ethiopia. Thus, this study assessed sleep quality and its associated factors among diabetes, hypertension, and heart failure patients.

## 2. Methods and Materials

### 2.1. Study Design and Area

An institutional-based cross-sectional study design was conducted at the Debre Markos Referral Hospital Chronic Illness Follow-up Clinic which is found in Debre Markos town from February 22, 2018, to April 6, 2018. There are 4 rooms in the clinic. A total of 4 nurses and 4 general practitioners were working in this clinic. The total number of diabetes, hypertension, and heart failure patients at Debre Markos Referral Hospital Chronic Illness Follow-up Clinic were 2368 (1052 DM patients + 494 heart failure patients + 822 hypertensive patients) [21].

### 2.2. Population

#### 2.2.1. Source Population

All diabetes, hypertension, and heart failure patients who are on follow-up in Debre Markos Referral Hospital Chronic Illness Follow-up Clinic were sources of population.

#### 2.2.2. Study Population

All diabetes, hypertension, and heart failure patients who are on follow-up in Debre Markos Referral Hospital Chronic Illness Follow-up Clinic during the data collection period were study populations.

### 2.3. Inclusion and Exclusion Criteria

#### 2.3.1. Inclusion Criteria

On follow-up patients who are diagnosed either hypertensive or having diabetes or heart failure and who are 18 years and above were included.

### 2.4. Exclusion Criteria

Diabetes, hypertension, and heart failure patients who have history of hospital admission in the past one month, who have any known comorbid diseases including sleep disorders and acute infection in the past one month, and who are seriously ill were excluded.

### 2.5. Sample Size Determination

Sample size for the first objective was calculated by using the single population proportion formula with a 95% confidence level, 4% margin of error, and proportion of poor sleepers among patients with heart failure. Proportion, which is 81.65%, was taken from a study conducted on sleep quality among heart failure patients at Jimma University Specialized Hospital Chronic Illness Follow-up Clinic in 2015 [16]. Based on these assumptions, the sample size was calculated as follows:

(1) $$\begin{array}{c} N=\frac{{\left(Z_{a/2}\right)}^{2}\left(p\right)\;\left(1-p\right)}{d^{2}}, \end{array}$$

where *N* is the sample size, *Z*_*a*/2_ = 1.96 (standardized normal distribution curve value for the 95% confidence interval), *p* = 0.8165 (proportion of poor sleeper among patients with heart failure), and *d* = 0.04 (degree of margin of error) = (((1.96)^2^ (0.8165) (0.1835))/(0.04)^2^) = 359.7 = ~360.

Sample size for the second objective which is calculated by STATCalc Epi Info version 7 is shown in Table 1.

The sample size which was calculated for the first objective was the largest one. Therefore, by adding 10% nonresponse rate of 360, the total sample size was 396.

### 2.6. Sampling Technique

The study utilized the stratified random sampling technique. Initially, patients were stratified into diabetes, hypertension, and heart failure based on their diagnosis. Then, study participants were selected by systematic sampling in every *k*^th^ value which is 6 from each stratum.

### 2.7. Study Variables

The dependent variable was sleep quality while independent variables were sociodemographic variables (sex, age, educational level, marital status, residence, occupation, monthly income, family size, weight, height, and BMI), disease characteristics (duration of disease since diagnosis, number of medication, adherence to medication, and perception towards prognosis of illness), individual factor: perception towards prognosis of illness, substance and alcohol use (coffee and tea use, smoking, chat chewing, uses of hashish and shisha, and alcohol drinking), and other factors (anxiety, depression, physical activity, and health education about sleep hygiene).

### 2.8. Data Collection Tool and Procedure

The data were collected by using a structured questionnaire which is adopted from previous research [4]. It has 3 parts. The first part asked about sociodemographic status of study participants. The second part measured sleep quality by the Pittsburgh Sleep Quality Index (PSQI) questionnaire which is a golden standard to measure sleep quality. It has 19 items with seven components. Component 1 is subjective sleep quality; Component 2 is sleep latency; Component 3 is sleep duration; Component 4 is habitual sleep efficiency; Component 5 is sleep disturbances; Component 6 is use of sleep medicine; and Component 7 is daytime dysfunction. Validity and reliability of the PSQI were checked in the Ethiopian population at Mizan Aman town, Southwest Ethiopia [22]. In this study, internal reliability of the seven PSQI components was Cronbach alpha 0.73 which is above the acceptable Cronbach alpha. The third part focused on factors that affect sleep quality of diabetes, hypertension, and heart failure patients which include anxiety, depression, physical activity, substance use, alcohol use, support from anyone, and health education about sleep hygiene.

Generalized Anxiety Disorder Item 7 (GAD-7) is a 7-item questionnaire, whose internal reliability in Ethiopia is 0.917 [61], which was used to screen anxiety.

Patient Health Questionnaire Item 2 (PHQ-2) is a 2-item questionnaire, whose internal reliability in Ethiopia is 0.92 [63], which was used to screen depression.

International Physical Activity Questionnaire Item 7 (IPAQ-7) is also a standardized questionnaire used to assess physical activity of patients with chronic illness. It is validated in the Ethiopian population [65].

All part of the questionnaire was prepared in the English version initially and translated into Amharic then back to English to check their consistency. Four bachelor holder nurses collected the data by face to face interview after the patients finish their visit. Height and weight were measured for nonpregnant and edematous patients during the data collection period by data collectors. To avoid repeated interview for patients with repeated visits during the data collection period, data collectors asked and verified the patients whether they are interviewed or not before.

### 2.9. Data Analysis

After data collection, completely collected data were entered into EpiData version 3.1 and exported to Statistical Product and Service Solution (SPSS) version 25 for analysis. Multivariable binary logistic regression was done by taking variables that have *P* value of ≤0.2 from bivariable logistic regression to identify factors associated with sleep quality. Additionally, to compare means of sleep quality among diabetes, hypertensive, and heart failure patients, Kruskal-Wallis with Games-Howell post hoc test was enrolled. This nonparametric test was used since the assumptions for the parametric test (one-way anova) failed.

### 2.10. Operational Definition

1. *Good Sleep Quality*. After calculating the global score of sleep quality, the global score of PSQI is five and below [19, 23]
2. *Poor Sleep Quality*. After calculating the global score of sleep quality, the global score of PSQI is above five [19, 23]
3. *Anxiety*. Study participants who scored 9 and above in the Generalized Anxiety Disorder Item 7 (GAD-7) questionnaire were categorized as having anxiety [24]
4. *Inactive*. Inactive are those individuals who do not meet criteria for minimally active or Health-Enhancing Physical Activity (HEPA) [25]
5. *Health-Enhancing Physical Activity*. It is a vigorous-intensity activity on at least 3 days achieving a minimum of at least 1500 Metabolic Equivalent- (MET-) minutes/week or 7 or more days of any combination of walking and moderate-intensity or vigorous-intensity activities achieving a minimum of at least 3000 MET-minutes/week [25]
6. *Depression Disorder*. Study participants who scored 3 and above in the Patient Health Questionnaire Item 2 (PHQ-2) questionnaire were categorized as positive for depression disorder while those who scored less than 3 were categorized negative for depression disorder [26]
7. *Minimally Active*. It is defined as 3 or more days of vigorous activity of at least 20 minutes per day or 5 or more days of moderate-intensity activity or walking of at least 30 minutes per day or 5 or more days of any combination of walking, moderate-intensity, or vigorous-intensity activities achieving a minimum of at least 600 Metabolic Equivalent- (MET-) min/week [25]

## 3. Result

### 3.1. Sociodemographic and Economic Characteristics

From a total of 396 respondents, 384 respondents with 97.7% response rate participated in this study. Among these, 179 (46.6%) were female, 152 (39.6%) were illiterate (unable to read and write), 241 (62.8%) were married, and 138 (35.9%) were farmers.The median age of respondents was 45 (IQR = 30), and 53 (13.8%) of the respondents were 65 and above years of age. From the total respondents, 151 (39.3%) of them lived in rural areas. Although 149 (38.8%) of the respondents had family size of more than four, above one-fifth of the total respondents 84 (21.9%) did not get any support from others. BMI was calculated for 371 nonpregnant and edematous respondents, and among these, above one-fifth of the respondents 78 (21%) were overweight (BMI ≥ 25 kg/m^2^) (Table 2).

### 3.2. The Pittsburgh Sleep Quality Index (PSQI) Subscale Scores and Level of Sleep Quality

#### 3.2.1. Rate of Overall Subjective Sleep Quality (Component 1)

**Figure 1:**
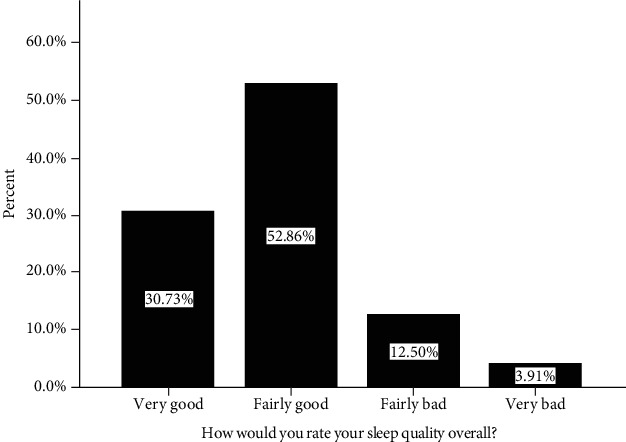
Rate of overall subjective sleep quality among patients with chronic illness at Debre Markos Referral Hospital Chronic Illness Follow-up Clinic, 2018.

From a total of 384 study participants, 63 (16.41%) of them rated their overall sleep quality as bad (Figure 1). More than one-third of the respondents 123 (32%) took more than 30 minutes to fall asleep, and 134 (34.9%) of the study participants slept less than 7 hours of duration with mean sleep duration of 7.09 (SD = 1.5). Only less than half of the participants 178 (46.4%) slept 85% and above of their time spent in bed. Majority 376 (97.9%) of the study participants did not use both prescribed and nonprescribed medication for their sleep disturbance. Fifty-eight (15.1%) of the total study participants reported that their sleep affects their day to day function (Table 3)

#### 3.2.2. Sleep Disturbance (Component 5)

From the total study participants, 288 (75%) reported that their sleep is disturbed by waking up in the middle of the night or early morning. Additionally, according to the report of 255 (66.4%) respondents, nocturia (having to get up to use the bathroom) was the other reason that disturbs sleep. Feeling too hot and bad dream were the other reasons for sleep disturbance reported by one-fifth of the respondents.

#### 3.2.3. Level of Sleep Quality

After summation of the seven components of PSQI, the mean PSQI global (total) score in this study was 5 (SD = 3.48). From 384 study participants, 140 (36.5%) had poor sleep quality.

### 3.3. Factors Associated with Sleep Quality

Variables which have an association with sleep quality at *P* value ≤ 0.2 in bivariable logistic regression were sex, age, marital status, educational level, occupation, residence, family size, perception towards prognosis of illness, alcohol drinking, anxiety, and depression. These were entered in multivariable logistic regression to identify factors associated with sleep quality. However, in multivariable logistic regression, only age, educational level, residence, perception towards prognosis of illness, and anxiety were associated with sleep quality at *P* value of 0.05. According to the result, patients whose age is 65 years and above were 6.48 times more likely to be a poor sleeper when compared with patients with 18-24 years of age (AOR = 6.48, 95%CI = 2.35‐18.57, *P* = 0.000). Similarly, patients whose educational level is certificate were 5.46 times more likely to be a poor sleeper than illiterate patients (AOR = 5.46, 95%CI = 1.30‐22.81, *P* = 0.020). Patients with chronic illness who lived in rural areas had poor sleep quality more likely than patients with chronic illness who lived in urban areas (AOR = 1.96, 95%CI = 1.07‐3.59, *P* = 0.028). Likewise, patients who have poor perception towards prognosis of their illness were 4.21 times more likely to be a poor sleeper when compared with patients who have good perception (AOR = 4.21, 95%CI = 1.94‐9.13, *P* = 0.001) (Table 4).

### 3.4. Difference of Sleep Quality among Diabetes, Hypertension, and Heart Failure Patients

The comparison of sleep quality among patients with diabetes, hypertension, and heart failure was executed with the Kruskal-Wallis test and Games-Howell post hoc test since it is not normally distributed and the variance is not equal (Levene's test (*F* (2, 381) = 5.09, *P* value = 0.007); Shapiro-Wilk test *P* value = 0.000). There was a statistically significant difference between patients with diabetes and hypertension, and diabetes and heart failure (*F* (2, 384) = 10.92, *P* = 0.004). Although difference of mean sleep quality is observed between patients with hypertension and heart failure, it is not statistically significant. Patients with diabetes have the lowest global PSQI score and best sleep quality.

## 4. Discussion

The quality of sleep especially in patients with chronic illness should be assessed since it affects cognitive, physical, and psychosocial health in a multidimensional way. Poor sleep quality negatively influences the self-care behavior of patients with chronic illness which is a key way to manage their illness, and this in turn impaired their functional outcome and prognosis of their illness. Thus, the purpose of this study was to assess the quality of sleep and associated factors among diabetes, hypertension, and heart failure patients aiming to enhance the management of chronic illness and improve the self-care behavior of patients.

The prevalence of poor sleep quality in this study was 36.5% (95% CI: 31.8%-41.1%). This is in line with the previous finding in Korea (38.4%) [27], Xuzhou City of China (33.6%) [17], Northwest Iran (38%) [28], and Korean University Hospitals (38%) [29].

However, the finding of the current study is lower than the finding of a study conducted in Jimma University Specialized Hospital, Southwest Ethiopia (81.65%) [16], North Central Nigeria (44%) [30], Northern Nigeria (68.7%) [31], Klang Valley, Malaysia (47.2%) [32], USA (80%) [14], Santa Catarina, Brazil (55.7%) [33], South India (64%) [34], and Tehran University of Iran (79%) [35]. The possible justification for this discrepancy could be the difference in the cutoff point of poor sleep quality, inclusion of patients with comorbid disease, difference in sampling technique and sample size, and difference in sociodemographic and cultural status. In addition to these, majority of patients use chat in the study conducted at Jimma University Specialized Hospital, Southwest Ethiopia. Since it affects the sleep latency and duration, it might be one reason for the discrepancy. The percentage of poor sleep quality among patients during their first visit may be high because of high expectation from the health care providers. This also might be another reason for the discrepancy of the results between the current study and the finding in Northern Nigeria.

In the current study, waking up at the middle of the night or early morning and nocturia (waking up to use the bathroom) were the most common reasons for disturbance of sleep. This is because these reasons decrease sleep duration because of frequent waking up and interrupted sleep cycle. This is in line with study conducted at Brazil, Malaysia, and Yazed City of Iran [32, 36, 37].

The odds of being a poor sleeper are increased when age increased. The current study showed that patients whose age are 65 years and above were six times more likely to be poor a sleeper compared with patients whose age is 18-24 years. The possible justification for this could be increment of sleep latency, frequent waking up, waking up early in the morning, and not having deep sleep. All these might be a result of deterioration of sleep/wake homeostasis and circadian rhythm in age increment. In age increment, the function of the hypothalamus which controls the cycle of sleep/wake homeostasis and circadian rhythm is decreased. This is in line with the finding of the study conducted in Northwest Iran [28] and Klang Valley, Malaysia [32].

In this study, the association between sleep quality and educational level is also observed. Patients who have a certificate were five times more likely to be a poor sleeper compared with those who did not read and write. Although the exact reason is unknown, the possible justification might be since individuals who have a certificate have different heavy tasks such as education for upgrading themselves, workload to increase income, and less satisfying occupation. This finding is supported by the earlier finding in Jimma University Specialized Hospital, Southwest Ethiopia [16], and Tehran University of Iran [35].

Additionally, patients who lived in rural areas were two times more likely to be a poor sleeper than patients who lived in urban areas. The possible justification for this could be since patients who lived in rural areas have uncomfortable, less satisfying living condition and poor health perception and management than patients who lived in urban areas. In addition, patients who lived in rural areas could have light sleep for a longer period of time since they sleep early.

This study also showed the association between sleep quality and perception towards prognosis of illness. Patients who have poor perception towards prognosis of their illness were four times more likely to be a poor sleeper compared with patients who have good perception towards prognosis of their illness. The possible justification for this might be decreased hope and being stressed regarding the prognosis of their illness among patients with poor perception. This finding is in line with the previous finding in USA [38].

Anxiety and sleep quality had also significant association. This study revealed that patients who have anxiety were four times more likely to be a poor sleeper compared with patients without anxiety. This is due to bidirectional relation of anxiety and sleep quality although it is difficult to know the direction of relation in this cross-sectional study. This finding is in line with the finding in Northwest Iran [28], Malaysia [32], and Japan [11].

As to the National Sleep Foundation, the recommended sleep duration for young adult and adult is 7-9 hours per day [2]. But in this study, over one-third of the study participants slept less than 7 hours which is lower than the recommendation.

### 4.1. Limitation of the Study

This study has its own limitations. Using a cross-sectional study design is one limitation. Because of the subjective nature of the questionnaire, recall bias may be present. In addition, this study assessed the quality of sleep for only three chronic illnesses.

## 5. Conclusion

In this study, over one-third of patients had poor sleep quality. Age, educational level, residence, perception towards prognosis of illness, and anxiety were factors associated with sleep quality. Waking up in the middle of the night or early morning and nocturia (waking up to use the bathroom) were the most common reasons that disturb sleep. There was difference of sleep quality between patients with diabetes and hypertension, and diabetes and heart failure.

## 6. Recommendations

Health care workers have to assess sleep quality of diabetes, hypertension, and heart failure patients in every visit; provide advice and education about sleep hygiene, elimination pattern, and anxiety; improve their perception towards the prognosis of their illness; and provide advice on how to improve sleep quality to patients who live in rural areas. In addition, they should manage and refer those patients with poor sleep quality to a psychiatrist and provide appropriate counseling. Assessment of sleep quality for every diabetes, hypertension, and heart failure patient in every visit should be incorporated in the care package.

## Figures and Tables

**Table 1:** Sample size for significant factors that affect sleep quality at Debre Markos Referral Hospital Chronic Illness Follow-Up Clinic, 2018.

S. No.	Factors associated with sleep quality	Total largest sample size from Epi Info STATCalc	AOR	Reference
1	Sex	80	4.058	Study conducted at India [23]
2	Depression	118	3.828	Study conducted at Northwest Iran [28]
3	Alcohol drinking	140	0.343	Study conducted at Korea University Hospitals [29]

AOR: adjusted odds ratio.

**Table 2:** Sociodemographic status of diabetes, hypertension, and heart failure patients at Debre Markos Referral Hospital Chronic Illness Follow-up Clinic, 2018 (*N*-384).

Variable	Category	Frequency	Percentage
Sex	Female	179	46.6
	Male	205	53.4
	Total	384	100

Age	18-24	52	13.6
	25-29	38	9.9
	30-34	30	7.8
	35-44	65	16.9
	45-64	146	38.0
	≥65	53	13.8
	Total	384	100

Educational level	Unable to read and write	152	39.6
	Able to read and write (informal school)	32	8.3
	Grades 1-8	53	13.8
	Grades 9-12	54	14.1
	Certificate	12	3.1
	Diploma and above	81	21.1

Marital status	Single	68	17.7
	Married	241	62.8
	Widow	53	13.8
	Divorced	22	5.7

Residence	Urban	233	60.7
	Rural	151	39.3

Occupation	Farmer	138	35.9
	Merchant	125	32.5
	Student	26	6.8
	Government or nongovernment employee	84	21.9
	Others (retired, no permanent job)	11	2.9

Monthly income (ETB)	≤1000	113	29.4
	1001-2000	84	21.9
	2001-3500	96	25
	>3500	91	23.7

Family size	≤4	235	61.2
	>4	149	38.8

Any support	Yes	300	78.1
	No	84	21.9

BMI	Underweight	38	10.3
	Normal	255	68.7
	Overweight	78	21
	Total	371	100

Note: monthly income was categorized based on quartile range; family size was based on mean; BMI was based on WHO weight classification for Ethiopia.

**Table 3:** The Pittsburgh Sleep Quality Index (PSQI) subscale scores and level of sleep quality among diabetes, hypertension, and heart failure patients at Debre Markos Referral Hospital Chronic Illness Follow-up Clinic, 2018 (*N* = 384).

PSQI subscale	Category	Frequency	Percent (%)
Time to fall a sleep Subscale of sleep latency (Component 2)	0-15 minutes	116	30.2
	16-30 minutes	145	37.8
	31-60 minutes	110	28.6
	>60 minutes	13	3.4

Sleep duration (Component 3)	≥7 hours	250	65.1
	6-7 hours	72	18.8
	5-6 hours	35	9.1
	Less than 5 hours	27	7

Habitual sleep efficiency (Component 4)	≥85%	178	46.4
	75-84%	108	28
	65-74%	39	10.2
	Less than 65%	59	15.4

Medication use for sleep (Component 6)	Not during the past month	376	97.9
	Less than once a week	3	0.8
	Once or twice a week	1	0.3
	Three or more times a week	4	1

Daytime dysfunction (Component 7)	Not during the past month	326	84.9
	Less than once a week	35	9.1
	Once or twice a week	10	2.6
	Three or more times a week	13	3.4

**Table 4:** Bivariable and multivariable logistic regression output on the association of sleep quality and factors, 2018 (*N* = 384).

Variable	Category	Sleep quality		COR	AOR	*P* value
		Poor	Good			
Sex	Female	72	107	1.36 (0.89-2.05)		
	Male	68	137	1		

Age	18-24	12	40	1	1	0.001
	25-29	9	29	1.03 (0.38-2.77)	0.99 (0.33-2.96)	
	30-34	10	20	1.66 (0.62-4.51)	3.12 (0.99-9.79)	
	35-44	22	43	1.70 (0.75-3.89)	3.10 (1.20-7.96)	
	45-64	60	86	2.33 (1.13-4.80)^∗^	4.32 (1.81-10.3)	
	≥65	27	26	3.46 (1.49-8.02)^∗^	6.48 (2.35-17.9)	

Educational level	Unable to read and write	57	95	1	1	0.001
	Able to read and write	16	16	1.66 (0.77-3.59)	3.25 (1.31-8.03)	
	Grades 1-8	25	28	1.49 (0.79-2.79)	5.40 (2.41-12.1)	
	Grades 9-12	17	37	0.77 (0.39-1.48)	2.57 (1.06-6.18)	
	Certificate	7	5	2.33 (0.70-7.69)	5.46 (1.30-22.8)	
	Diploma and above	18	63	0.47 (0.26-0.88)^∗^	1.66 (0.70-3.88)	

Marital status	Single	9	50	1		
	Married	88	153	1.59 (0.88-2.90)		
	Widowed	25	28	2.48 (1.15-5.31)^∗^		
	Divorced	9	13	1.92 (0.70-5.26)		

Residence	Urban	72	161	1	1	
	Rural	68	83	1.83 (1.19-2.80)^∗^	1.96 (1.07-3.59)	0.028

Occupation	Farmer	61	77	1		
	Merchant	46	79	0.74 (0.45-1.20)		
	Student	5	21	0.30 (0.10-0.84)^∗^		
	Employee	23	60	0.48 (0.27-0.86)^∗^		
	Others (retired, no job)	5	6	1.05 (0.30-3.61)		

Family size	≤4	77	158	1		
	>4	63	86	1.50 (0.98-2.29)		

Perception to prognosis of illness	Good	68	172	1	1	0.001
	Fair	49	56	2.21 (1.38-3.56)^∗^	1.74 (1.02-2.96)	
	Poor	23	16	3.63 (1.81-7.30)^∗^	4.21 (1.94-9.13)	

Anxiety	Have anxiety	67	56	3.08 (1.97-4.81)^∗^	3.69 (2.19-6.20)	<0.000
	Have no anxiety	73	188	1	1	

Depression	Have depression	15	7	4.06 (1.61-10.22)^∗^		
	Have no depression	125	237	1		

Notes: Hosmer and Lemeshow test = 0.449; ^∗^significant variables at *P* value < 0.05 in bivariable logistic regression.

## Data Availability

The dataset will not be shared in order to protect the participants' identities but is available from the corresponding author on reasonable request.

## Abbreviations

AOR: Adjusted odds ratio
BMI: Body mass index
CDC: Center of Disease Control and Prevention
COD: Crude odds ratio
DM: Diabetes mellitus
ETB: Ethiopian birr
GAD: Generalized Anxiety Disorder
HEPA: Health-Enhancing Physical Activity
IPAQ-7: International Physical Activity Questionnaire Item 7
MET: Metabolic Equivalent
PHQ-2: Patient Health Questionnaire Item 2
PSQI: Pittsburgh Sleep Quality Index
SD: Standard deviation
SPSS: Statistical Product and Service Solution
USA: United States of America.
